# Brain State-Dependent Brain Stimulation

**DOI:** 10.3389/fpsyg.2018.02108

**Published:** 2018-11-01

**Authors:** Til O. Bergmann

**Affiliations:** ^1^Department of Neurology and Stroke, Hertie Institute for Clinical Brain Research, University of Tübingen, Tübingen, Germany; ^2^Institute of Medical Psychology and Behavioral Neurobiology, University of Tübingen, Tübingen, Germany; ^3^Deutsches Resilienz Zentrum, University Medical Center Mainz, Mainz, Germany

**Keywords:** transcrancial magnetic stimulation (TMS), transcranial alternating current stimulation (tACS), transcranial direct current stimulation (tDCS), real-time, EEG

For the last 30 years, non-invasive brain stimulation (NIBS) approaches, using transcranial magnetic stimulation (TMS) and transcranial direct or alternating current stimulation (TCS), have treated the brain as a black box, ignoring its internal state at the time of stimulation. While inter-individual variability is long known to undermine the replicability of NIBS effects (Figure 1A), intra-individual variability across and within sessions has only recently gained attention (Ziemann and Siebner, 2015). NIBS effects are state-dependent on a time scale of minutes to hours, depending on the immediate history of neural activity (Silvanto et al., 2008) and synaptic plasticity (Ziemann and Siebner, 2008; Karabanov et al., 2015). However, brain states also change on the time scale of seconds to milliseconds, as neurons are heavily influenced by the temporospatial dynamics of spontaneous network activity, governed by rhythmic fluctuations in neural excitability (Buzsáki and Draguhn, 2004; Schroeder and Lakatos, 2009) under the control of ascending neuromodulatory systems and thalamo- and cortico-cortical projections (Lee and Dan, 2012; Harris, 2013; Zagha and McCormick, 2014). Frequency, amplitude, and phase of neuronal oscillations constitute transient local, network, or even global brain states that not only determine the fate of incoming sensory stimuli (VanRullen and Koch, 2003; Sadaghiani et al., 2010), but also affect both the immediate (“online”) neuronal response to NIBS and the subsequent after-effects (“offline”) resulting from NIBS-induced synaptic changes. It has therefore been suggested to not only optimize NIBS protocols based on neuroimaging data to account for individual differences in functional neuroanatomy (Bergmann et al., 2016; Thut et al., 2017) but also to take the current oscillatory brain state into account (Bergmann et al., 2016; Karabanov et al., 2016; Zrenner et al., 2016). Technical advances allow to assess ongoing multi-channel EEG data in real-time (Bergmann et al., 2012b; Thies et al., 2018; Zrenner et al., 2018) and modify stimulation parameters on the fly (Habibollahi Saatlou et al., 2018) to apply *brain state-dependent brain stimulation* (BSDBS).

**Figure 1:**
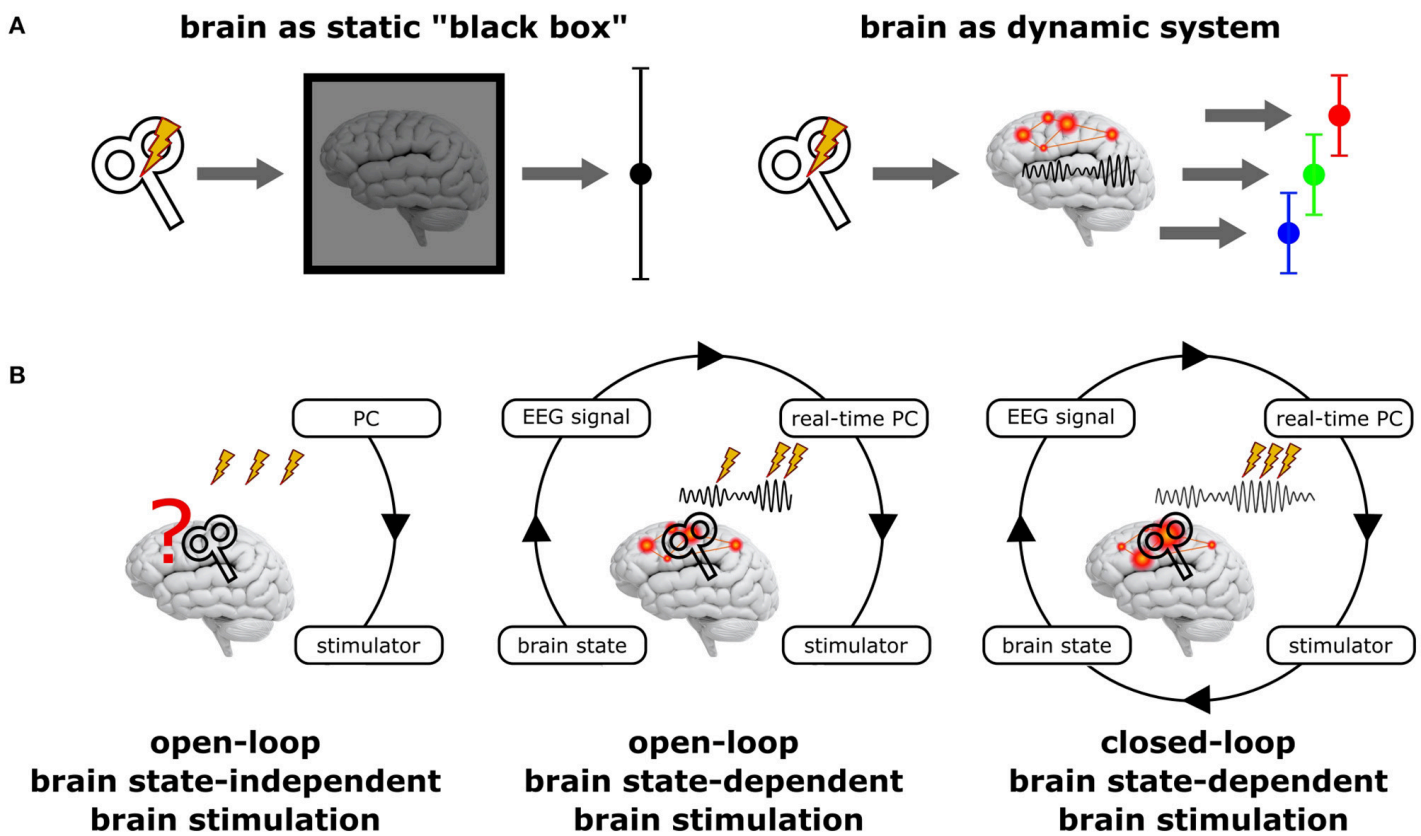
Principal scenarios of brain stimulation with respect to the current brain state. **(A)** Standard NIBS approaches treat the brain as a static “black box” (left), disregarding its variable internal state and may hence result in highly variable stimulation effects. In contrast, treating the brain as the dynamic system it actually is (right) may reveal very different (state-dependent) effects, but each of them being more homogenous. **(B)** *Open-loop brain state-independent brain stimulation* neglects the current brain state; no neuroimaging method and no real-time system is necessary to control the stimulation. *Open-loop brain state-dependent brain stimulation* (BSDBS) uses concurrent neuroimaging (e.g., EEG) and real-time signal analysis to monitor the current brain state and to adjust and trigger brain stimulation accordingly, however, without systematically changing the monitored target brain state (e.g., TMS triggered by the amplitude or phase of a certain EEG oscillation to assess state-specific corticospinal excitability but without considerable effect on the monitored oscillation). *Closed-loop BSDBS* additionally requires that the monitored brain state is actually changed by the stimulation, allowing to control the expression of a certain brain state.

## Open-loop vs. closed-loop BSDBS

BSDBS is often equated with *closed-loop stimulation*, which is not justified in most cases (Figure 1B). A closed-loop circuit in the strict sense continuously monitors a specific parameter of a system (e.g., a certain state-space of the brain) and adjusts a control signal (e.g., brain stimulation) accordingly to achieve and maintain a desired set-point of the monitored parameter (e.g., a specific brain state), just like a thermostat measures room temperature and regulates hot water influx to a radiator in order to achieve and maintain a predefined room temperature. But if the control signal has no effect on the monitored parameter (e.g., if brain stimulation does not effectively alter the monitored brain state), the loop remains open, even though the stimulation was applied in a brain state-dependent fashion.

## Applications for BSDBS

While BSDBS may have the potential to reduce the variability of NIBS effects (Figure 1A), it first and foremost provides a unique opportunity to study the neurophysiology and function of brain states, in particular neuronal oscillations. NIBS in general can be used *online* to *quantify* network properties (such as cortical excitability or connectivity), *interfere* with task-related neuronal activity (to impair behavioral performance), or *modulate* the level and timing of neuronal activity (e.g., to entrain neuronal oscillations and affect associated cognitive function); alternatively, NIBS can also be used *offline* to change synaptic efficacy, inducing LTP- and LTD-like changes in cortical excitability and connectivity (for a conceptual introduction to NIBS approaches see Bergmann et al., 2016). Importantly, all these strategies can be also employed in a brain state-dependent manner to study neuronal oscillations. Real-time EEG-triggered TMS has been used to *quantify* the excitability profile of specific oscillations: corticospinal excitability is larger during the peak (*up-state*) than the trough (*down-state*) of the sleep slow oscillation (<1 Hz) (Bergmann et al., 2012a), whereas it is increased during the trough compared to the peak of the 8–14 Hz sensorimotor mu-alpha rhythm (Zrenner et al., 2018) and is positively related to mu-alpha amplitude (Thies et al., 2018). TMS may also be used to *interfere* with information processing that is time-locked to specific oscillatory events or phases to probe their causal role for a cognitive function, such as memory reactivation during slow oscillation-spindle-ripple coupling (Staresina et al., 2015) or visual processing during alpha-gamma coupling (Jensen et al., 2014). Eventually, BSDBS may also be used to *modulate*, i.e., up- and down-regulate neuronal oscillations via the repeated time-locked stimulation of specific oscillatory phases, in analogy to the EEG-triggered auditory closed-loop modulation of sleep slow oscillations (Ngo et al., 2013). Recent TACS studies demonstrated feasibility of semi-closed-loop BSDBS (with brain state monitoring being interrupted during TACS application due to massive stimulation artifacts) for slow oscillations (Ketz et al., 2018) and spindles (Lustenberger et al., 2016), as well as tremor modulation (using peripheral accelerometry as a proxy for the neuronal tremor signal) (Brittain et al., 2013). But also *offline* BSDBS has been developed: Inspired by seminal work in rodents demonstrating that LTP- and LTD-like plasticity can be induced by bursts of electric stimulation timed to the peak or trough of the ongoing hippocampal theta oscillation (Huerta and Lisman, 1995, 1996), Zrenner et al. (2018) recently used EEG-triggered TMS bursts to induce phase-dependent plasticity in the human motor cortex with respect to the sensorimotor mu-alpha rhythm (Zrenner et al., 2018). Repeatedly targeting the more excitable oscillatory phase, one may tap into the same neural mechanism that underlies the proposed role of cross-frequency phase-amplitude coupling (PAC) in synaptic plasticity (Bergmann and Born, 2018).

## Challenges and future perspectives for BSDBS

So far BSDBS mainly relies on EEG due to its high temporal resolution, the ease of application and real-time data extraction, and its principal compatibility with NIBS. However, similar (MEG) or complementary (fMRI or fNIRS) neuroimaging techniques should be employed for BSDBS in the future, even though their combination with NIBS is more challenging (Bergmann et al., 2016). The accessibility of deep brain structures together with its excellent spatial resolution and whole brain coverage renders fMRI a highly promising tool to extract more complex brain states, e.g., using multi-voxel pattern classification, even though its low temporal resolution prevents a direct assessment of most neuronal oscillations. Regarding real-time signal analysis, the major challenge is to improve oscillatory brain-state extraction by developing better temporal and spatial filters, and more robust signal forecasting, which can be particularly demanding for oscillations with low signal-to-noise-ratio, non-sinusoidal waveforms, and high variability in amplitude and frequency over time. Also the spatial component of brain states and the adaptation of stimulation parameters should be considered. Automated robotic coil navigation (Harquel et al., 2017) or novel multi-channel coils (Koponen et al., 2018) principally allow to select stimulation sites in real-time, e.g., to follow traveling waves or to target different network nodes. Also NIBS intensity or frequency can be adapted online, e.g., to compensate spontaneous fluctuations in excitability or oscillatory frequency. Yet, the most important challenge will be to develop truly closed-loop BSDBS (Figure 1B) that allows to continuously monitor the brain signals of interest, while concurrently applying NIBS to achieve and maintain the desired brain state and to control perception or behavior. The successful real-time removal of TMS/TCS stimulation artifacts (Walter et al., 2012; Herring et al., 2015; Rogasch et al., 2017) and EEG correlates of multisensory co-stimulation during TMS (Herring et al., 2015; Conde et al., in press) and TCS (Schutter, 2016; Herring et al., 2018) is therefore a core developmental goal for the near future.

BSDBS is still in its very early stage, and many exciting applications yet remain to be uncovered. Importantly, to exploit the full potential of this novel technique, it needs to be applied in a hypothesis-driven manner, with a decent neurophysiological understanding of the target brain state, and carefully adapted to the research question at hand. It should not be considered as a new standard formula to improve any NIBS setup, but as an important step toward a higher degree of flexibility, specificity, and precision in NIBS.

## Author contributions

The author confirms being the sole contributor of this work and has approved it for publication.

### Conflict of interest statement

The author declares that the research was conducted in the absence of any commercial or financial relationships that could be construed as a potential conflict of interest.
